# Characterization of Closed Head Impact Injury in Rat

**DOI:** 10.1155/2015/272976

**Published:** 2015-09-16

**Authors:** Yi Hua, Praveen Akula, Matthew Kelso, Linxia Gu

**Affiliations:** ^1^Department of Mechanical and Materials Engineering, University of Nebraska-Lincoln, Lincoln, NE 68588-0656, USA; ^2^Department of Pharmacy Practice, University of Nebraska Medical Center, Omaha, NE 68198-6045, USA

## Abstract

The closed head impact (CHI) rat models are commonly used for studying the traumatic brain injury. The impact parameters vary considerably among different laboratories, making the comparison of research findings difficult. In this work, numerical CHI experiments were conducted to investigate the sensitivities of intracranial responses to various impact parameters (e.g., impact depth, velocity, and position; impactor diameter, material, and shape). A three-dimensional finite element rat head model with anatomical details was subjected to impact loadings. Results revealed that impact depth and impactor shape were the two leading factors affecting intracranial responses. The influence of impactor diameter was region-specific and an increase in impactor diameter could substantially increase tissue strains in the region which located directly beneath the impactor. The lateral impact could induce higher strains in the brain than the central impact. An indentation depth instead of impact depth would be appropriate to characterize the influence of a large deformed rubber impactor. The experimentally observed velocity-dependent injury severity could be attributed to the “overshoot” phenomenon. This work could be used to better design or compare CHI experiments.

## 1. Introduction

Traumatic brain injury (TBI) is the leading cause of mortality and morbidity in the United States, which affects over 1.7 million Americans each year [1]. It leads to long-term disability in cognition, sensorimotor function, and personality [2]. To study the mechanisms of TBI, the rat closed head impact (CHI) is commonly used for replicating the trauma events [3–9]. In a typical CHI procedure, the rat is fully anesthetized and secured in a stereotactic frame. An impact load is delivered directly to the intact skull through a pneumatically driven impactor. Compared with other animal models such as fluid percussion [10] and controlled cortical impact (CCI) [11], the CHI model avoided performing the craniotomy and thus the risk of operation-induced inflammation. The CHI model has been used for investigating the cortical tissue loss [3, 4], acute subarachnoid hemorrhage [5], diffuse axonal injury [6], blood-brain barrier dysfunction [7, 8], and concussion [9]. Nevertheless, various control parameters (e.g., impact depth, velocity, and position; impactor diameter, material, and shape) were employed by different research groups, making it hard to compare these results. In addition, the internal brain response variables could be documented in more detail. To address these shortcomings, it is crucial to establish the linkage between external impact parameters and intracranial responses.

In this work, the sensitivities of intracranial responses to various impact parameters in the CHI model were systematically investigated. A three-dimensional (3D) finite element (FE) rat head model with anatomical details was developed from medical images. An orthogonal experimental design was implemented for carrying out eight computational experiments to correlate the regional brain mechanics with impact controls including impact depth, velocity, and position, as well as impactor diameter, material, and shape.

## 2. Finite Element Modeling

**Figure 1:**
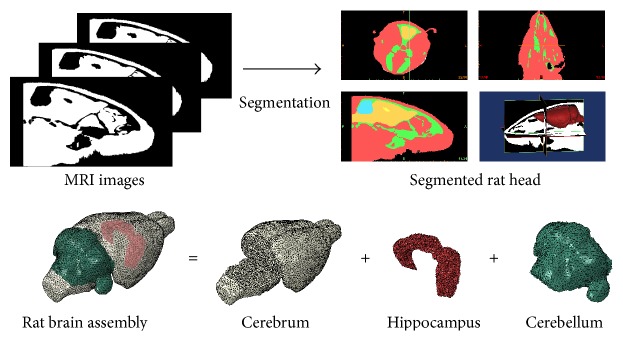
Finite element discretization of the adult male Sprague-Dawley rat head.

A 3D FE rat head model was generated from the high-resolution magnetic resonance imaging (MRI) datasets of an adult male Sprague-Dawley rat weighing about 360 g, as shown in Figure 1. The brain MRI has an isotropic resolution of 256 × 256 × 256 pixels, for a field view of 30 mm in all three directions. The image data were segmented into three main brain regions: cerebrum, hippocampus, and cerebellum. The segmentation was realized using the 3D image analysis algorithm implemented in Mimics (Materialise, Inc., Leuven, Belgium). The segmented brain model was then imported into HyperMesh (Altair Engineering, Inc., MI, USA) and meshed as a triangular surface mesh (S3R). A volume mesh with 4-noded tetrahedral elements (C3D4) was generated from this surface mesh. The rat skull was created by offsetting a layer of wedge elements (C3D6) above the outer surface of the brain by 0.16 mm [12]. A mesh convergence test was conducted and the minimum mesh size of 0.4 mm was chosen. At this resolution, the rat head model consisted of a total of 1,107,183 tetrahedral elements and 14,898 prism elements for the brain and skull, respectively.

The rat brain was assumed to be a linear viscoelastic material with a decay constant of 20 ms [13]. For the cerebrum, a short-term shear modulus of 1.72 kPa and a long-term shear modulus of 0.51 kPa were assumed. This assumption was based on the indentation test results obtained from the adult rat brain as reported by Gefen et al. [14]. The cerebellum had a short-term modulus of 1.2 kPa and a long-term modulus of 0.36 kPa [15], while the same parameters for the hippocampus were 4.06 kPa and 0.61 kPa, respectively [16]. The rat skull was modeled as a homogeneous linear elastic isotropic material and Young's modulus and Poisson's ratio were assumed as 6 GPa and 0.3, respectively [17]. A summarization of the material properties is described in Table 1.

**Figure 2:**
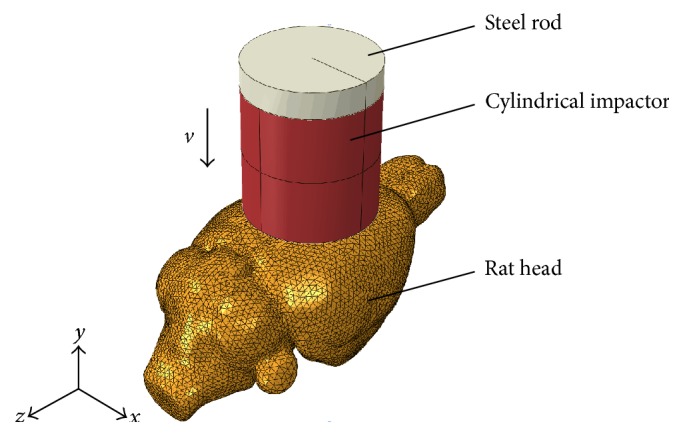
Isometric view of the closed head impact model.

To replicate the experimental CHI procedure, a cylindrical impactor, which connected to the bottom surface of a steel rod, was positioned perpendicular to the dorsal surface of the rat skull (Figure 2). A linearly ramping displacement was enforced onto the steel rod to achieve the prescribed impact depth and velocity. Due to variations in impactor geometries, materials, and positions, the prescribed conditions will result in different indentation behaviors. The interactions between the impactor and skull as well as between the brain and skull were modeled through penalty contact algorithm with tangential sliding and hard contact normal behavior. The nodes on the bottom surface of the skull were constrained in all six degrees of freedom to avoid rigid body translation. The FE model was solved using the nonlinear transient dynamic procedure Abaqus/Explicit (Dassault Systems Simulia Corp., RI, USA).

## 3. Design of Computational Experiments

A six-factor two-level orthogonal experimental design was implemented to systematically investigate the sensitivities of intracranial responses to various impact parameters in three different brain regions: cerebrum, hippocampus, and cerebellum. The six factors studied were the prescribed impact depth (A), velocity (B), and position (C), as well as impactor diameter (D), material (E), and shape (F). The baseline level of these factors was selected from the typical CHI procedures, including an impact depth of 1 mm, velocity of 3 m/s, central position around the midline between bregma and lambda, nylon impactor with a diameter of 6 mm, and a flat end. The second level of the quantitative factors, that is, impact depth, velocity, and impactor diameter, was double over the baseline. For the qualitative factors, the second level was selected as lateral impact position over the left parietal bone between bregma and lambda, steel impactor material, and convex end. An L_8_(2)^7^ orthogonal array from the module of Statistica (Version 10.0) was adopted to implement the multifactor combination. The assignment of six factors and their selected levels in the array was depicted in Table 2.

## 4. Results

### 4.1. Model Verification

**Figure 3:**
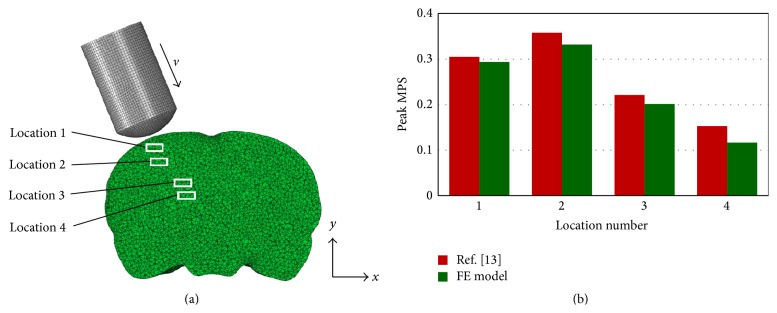
Verification of rat head model with [13]. (a) Coronal view of rat head subjected to controlled cortical impact and (b) peak MPS comparisons at four different locations of the brain.

The published CCI injury data [13] were used to verify the FE model. To simulate the cortical impact, a 7 mm diameter craniotomy was created on the left skull. The impactor shape and impact direction were accurately defined according to the settings in the cited publication. The relative position between the impactor and brain is shown in Figure 3(a). The impact depth and velocity were assumed to be 1.5 mm and 4 m/s, respectively. The peak maximum principal strain (MPS) was extracted at four different locations of the brain (Figure 3(a), locations 1–4), corresponding to the superior cortex, deep cortex, hippocampus, and thalamus as measured in [13].

Comparative results are shown in Figure 3(b). The peak MPS predicted by the FE model agreed well with the published data. The maximum deviation between the FE model and [13] was 23.8% at location 4, while the deviations at other locations were less than 9.0%. Moreover, in the FE model, location 2 experienced 13.0% higher peak MPS than location 1 although location 1 was more close to the impact site. This was consistent with the finding in [13], which measured a 17.4% higher peak MPS at deep cortex compared to the superior cortex.

### 4.2. Sensitivity Studies

**Figure 4:**
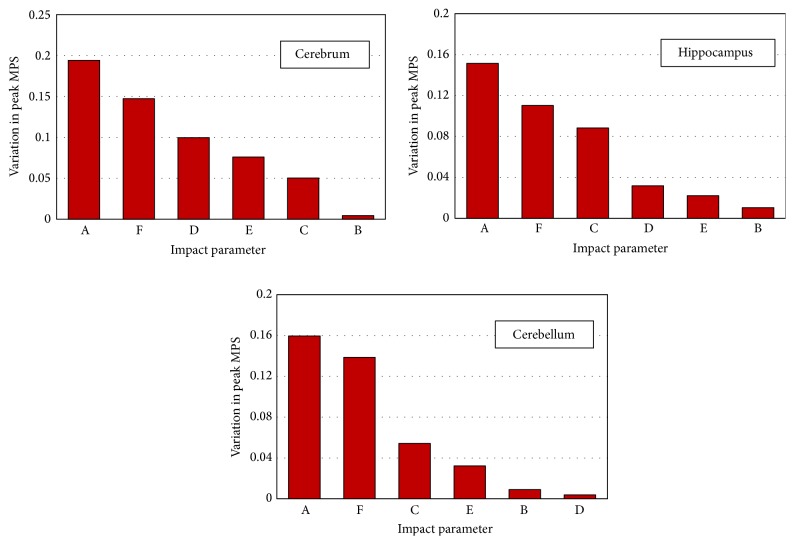
Pareto chart of region-specific biomechanical responses to external impact parameters. A: impact depth; B: impact velocity; C: impact position; D: impactor diameter; E: impactor material; F: impactor shape.

**Figure 5:**
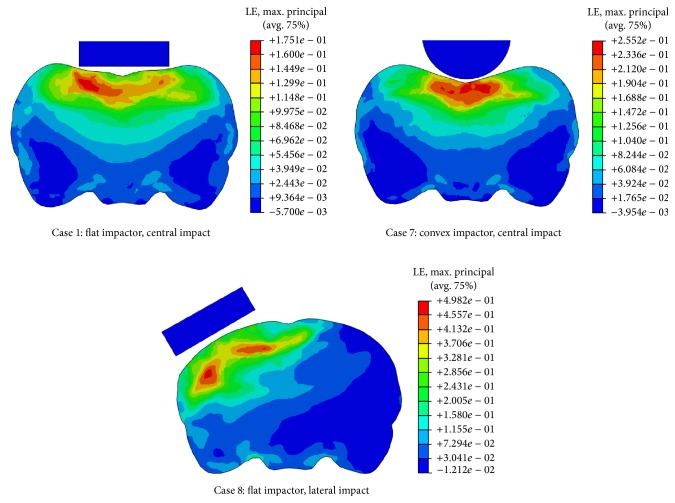
Contour plots of maximum principal strain (MPS) on a coronal plane of the rat brain.

The predicted MPS was used to characterize the brain responses. The peak MPS in the cerebrum, hippocampus, and cerebellum for all eight cases are listed in Table 2. The range analysis, which assumes that the influence of other factors on the result is balanced when analyzing the impact of a specific factor, was performed to quantify the significance level of each factor as shown in Table 3. The *K*_*i*_ value of a factor was the average of four values of peak MPS for level *i* listed in Table 2, and the range value *R* for each factor was the difference between *K*_*i*_ values of the two levels. A larger *R* indicates that the corresponding factor plays a more important role in the brain responses. The Pareto chart (Figure 4), based on the magnitude of range value *R*, has shown that impact depth and impactor shape were the two leading factors affecting biomechanical responses of the brain regardless of regions. For example, varying the impact depth from 1 mm to 2 mm produced an increase of peak MPS in the cerebrum of 0.1941, in the hippocampus of 0.1514, and in the cerebellum of 0.1595. As a flat impactor was changed to a convex one, the peak MPS decreased by 0.1473, 0.1102, and 0.1386 in the cerebrum, hippocampus, and cerebellum, respectively. Moreover, the high strains induced by the flat impactor were approximately parallel to the bottom rim of the impactor, while those induced by the convex one were concentrated along the axial line (Figure 5, Cases  1 and 7).

Following impact depth and impactor shape, impactor diameter ranked as the third most important factor for the calculated peak MPS in the cerebrum. However, impactor diameter had much less effect in the hippocampus and cerebellum. For example, the peak MPS in the cerebrum increased by 0.0997 when impactor diameter increased from 6 mm to 12 mm, compared to a limited increase of 0.0318 and 0.0038 in the hippocampus and cerebellum, respectively.

Impact position seems to have a uniform effect on the biomechanical responses in all three brain regions. Changing the impact position from central to lateral, the peak MPS in the cerebrum, hippocampus, and cerebellum increased by 0.06 ± 0.02. A coronal view of the rat brain exhibited totally different strain patterns when the impact position was changed (Figure 5, Cases  1 and 8). It is observed that a lateral impact induced higher MPS to the ipsilateral side of the brain and the strain magnitude in most of the region of the contralateral side was almost zero. In contrast, a similar strain gradient was found on both sides of the brain for the central impact.

Only small variations were found due to variations in impact velocity and impactor material on the predicted peak MPS in all three brain regions. Since impact depth, impactor shape, impactor diameter, and impact position could affect the intracranial responses both qualitatively and quantitatively, they are most critical when designing appropriate CHI models.

## 5. Discussion

In this work, the intracranial responses to various impact parameters in the CHI model were systematically investigated using the 3D FE rat head model. The peak MPS was chosen as the response variable since previous investigations have demonstrated that regions with higher MPS correlated well with the brain injury severity including contusion volumes and the percentage of neuronal cell loss [18, 19]. Our model was first verified by previously published CCI injury data [13] and good agreement has been achieved (Figure 3). It is observed that the peak MPS predicted by the FE model was generally lower than the published results. This could be attributed to the difference in weight and size of the rat which we used. The anatomy and geometry of the brain used in this work were taken from the rat weighing about 360 g, which was larger than that of 250–300 g in [13]. A recent study has demonstrated that increasing the size of rat brain would decrease the magnitude of predicted strain under impact loading [20].

Following model verification, an orthogonal experimental design was then used to quantify the significance levels of six impact parameters on the brain response. Our results (Figure 4) have shown that the prescribed impact depth is the leading factor affecting intracranial MPS responses in CHI. A larger impact depth could result in more severe axonal damage as well as increased permeability of the blood-brain barrier in the rat brain [7, 12]. This reinforced our finding that the intracranial peak MPS might serve as an index for the brain injury severity. On the contrary, the impact depth itself is not a reliable index based on observations that the impact-induced peak brain MPS could vary 103.2% in the cerebrum, 85.7% in the hippocampus, and 214.2% in the cerebellum under the same prescribed impact depth at 2 mm.

**Figure 6:**
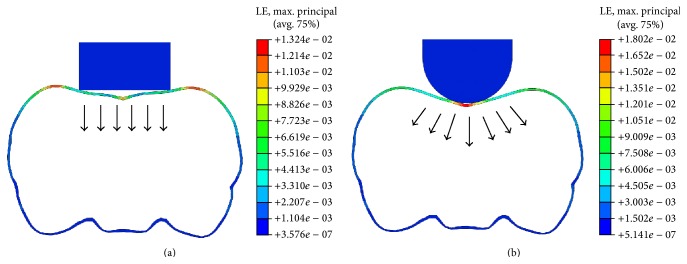
A coronal view of skull deformation for (a) flat impactor and (b) convex impactor.

Under the same boundary constrains, the convex impactor reduced the intracranial peak MPS compared to the flat one: that is, *K*_2_ < *K*_1_ (column F in Table 3). However, it induced strain concentration within the brain (Figure 5, Cases  1 and 7), associated with the severity of TBI, and a stress concentration in the skull, leading to higher incidence of skull fracture (Figure 6). This is why flat impactors were commonly used in documented studies to induce the diffuse injury within the brain and to reduce the skull fracture [3, 5–7, 9]. In addition, the impactor diameter in the CHI tests is generally larger than 6 mm [3, 6, 7, 9], whereas it is less than 5 mm in the CCI tests [11, 15]. An increase in impactor diameter could substantially increase tissue strains in the cerebrum, located directly beneath the impactor; however, the strains in the hippocampus and cerebellum seem to be not sensitive to this impact parameter.

**Figure 7:**
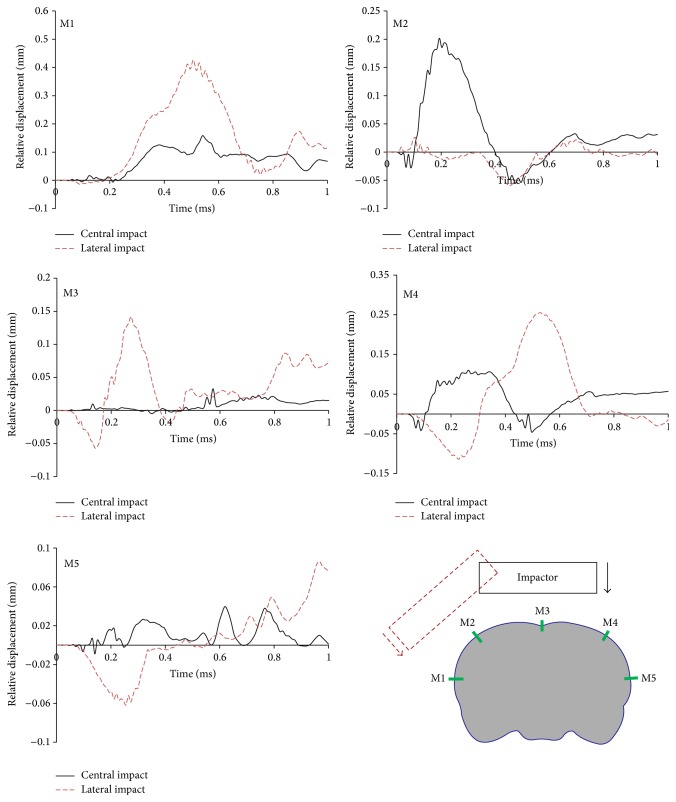
Relative displacement at five mark locations (M1–M5) along the brain/skull interface.

Impact position is found to be crucial in determining the extent and location of tissue injury in rat brain. Our results (Table 3) show that lateral impact induced higher peak MPS in all three brain regions compared to the central impact. Moreover, the high strains induced by the lateral impact were mainly focused on the ipsilateral side of the brain, while those induced by the central impact were more diffusely located on both sides (Figure 5, Cases  1 and 8). This is consistent with the clinical observation that lateral impact inflicts primarily unilateral cortical damage, whereas central impact causes bilateral cortical alterations [3, 7]. Furthermore, the lateral impact is able to induce a larger relative movement between the brain and skull, which contributes to the subarachnoid hemorrhage (SAH), a common cerebrovascular event following CHI [5, 6]. The relative displacement at five marked locations (M1–M5) along the brain/skull interface was compared between central and lateral impacts (Figure 7). The maximum relative displacement was 0.43 mm at location M1 subjected to lateral impact and 0.20 mm at location M2 subjected to central impact. This indicates that SAH is more likely to occur on the ipsilateral side of the brain. However, the relative displacement induced by the lateral impact was minimal at location M2. This is due to the fact that this location is along the midline of the lateral impactor, which constrained the relative motion between the brain and skull. A similar observation existed at location M3, along the midline of the central impactor, which limited the relative skull/brain displacement. This implies that a properly designed impact position is able to guide SAH to target a specific region of interest.

The effect of impactor material on the intracranial responses is found to be very limited. This indicates that the brain injury severity might not be sensitive to the impactor materials. However, a softer impactor is commonly recommended for the CHI to avoid skull fracture [3, 9]. To examine the significance of impactor materials on skull fracture using von Mises criteria, the peak von Mises stress in the rat skull was obtained for two impactor materials (e.g., steel and nylon). It is found that the peak stress decreased only by 1.87% when the impactor material changed from steel to nylon, indicating the almost same probability of skull fracture. Careful attention should be paid when the impactor is made of an extremely soft material such as rubber [4]. One more simulation was conducted using baseline data except the impactor materials. The rubber impactor-induced peak von Mises stress was 36.1% less than the steel one. It should be noted, though, that such reduction of skull stress is caused by the insufficiency of indentation depth. We measured a 1 mm shortening of the rubber impactor at the time of maximum indentation. This indicated that the actual indentation depth was 1 mm instead of the prescribed 2 mm impact depth, leaving the rat brain to be less injured. It is suggested to calibrate the prescribed impact depth or monitor the actual indentation depth when adopting a very soft impactor in the CHI tests.

It is interesting to find that variations in impact velocity induced only small variations on peak MPS in all three brain regions. On the other hand, a wide range of impact velocities are used in different laboratories with the goal to induce desired injury levels [6, 8]. This inconsistency could be attributed to the “overshoot” phenomenon which widely exists in the pneumatically driven impact devices [21]. Overshoot is referred to as the maximum transient displacement of the impactor tip that exceeds the predefined impact depth. We have captured the trajectory of the impactor tip of a commercially available pneumatic device TBI-0310 Impactor (Precision Systems and Instrumentation) using a high-speed camera (Photron SA 1.1). It was observed that overshoot was positively correlated with the impactor velocity. For a predefined impact depth of 3 mm, the device produced a 0.4 mm (13%) overshoot at an impact velocity of 2 m/s, while the overshoot was substantially increased to 1.5 mm (50%) at 5 m/s. This overshoot phenomenon is not considered in our computational models, which might underestimate the role of impact velocity. In addition, the rat skull was modeled as a uniform thin layer, rather than geometrical details such as varied thickness.

The brain tissue was simplified as an isotropic homogeneous material, rather than a site-dependent white and gray matter combination including a dense network of blood vessels, cellular structure, or differing cell types such as glial cells which are highly responsive to brain injury [22–24]. The exclusion of these elements might alter the magnitude of brain dynamics. Moreover, the rat brain was meshed using tetrahedral elements. Although this element type makes meshing complex geometries easier, it tends to exhibit a stiffer response when compared to quadrilateral or hexahedral meshes because of shear/volumetric locking. Despite these simplifications, the present work demonstrated the significance level of six input parameters in terms of the brain MPS, which may have significant clinical implications for brain injury. This work can be used to provide a fundamental understanding of the impact of CHI designs on the brain and to better design a site- and severity-specific rodent CHI model.

## Figures and Tables

**Table:** (a) Elastic material properties

Component	Density (kg/m^3^)	Young's modulus (MPa)	Poisson's ratio (/)
Skull	1710	6000	0.3

**Table:** (b) Viscoelastic material properties

Component	Density (kg/m^3^)	Short-term shear modulus (kPa)	Long-term shear modulus (kPa)	Decay constant (ms)
Cerebrum	1040	1.72	0.51	20
Cerebellum	1040	1.20	0.36	20
Hippocampus	1040	4.06	0.61	20

**Table 2:** Assignment of six factors and their selected levels in the L_8_(2^7^) orthogonal array and predicted peak maximum principal strain (MPS) in the cerebrum, hippocampus, and cerebellum.

Case number	Factors							Predicted peak MPS		
	A	B	C	D	E	F		Cerebrum	Hippocampus	Cerebellum
1	1	3	Central	6	Steel	Flat		0.1775	0.1466	0.1535
2	1	3	Lateral	6	Nylon	Convex		0.2283	0.1593	0.0326
3	1	6	Central	12	Steel	Convex		0.2061	0.0912	0.0494
4	1	6	Lateral	12	Nylon	Flat		0.4080	0.2993	0.1261
5	2	3	Central	12	Nylon	Flat		0.6192	0.3644	0.3890
6	2	3	Lateral	12	Steel	Convex		0.3744	0.3080	0.1238
7	2	6	Central	6	Nylon	Convex		0.3047	0.2204	0.1976
8	2	6	Lateral	6	Steel	Flat		0.4982	0.4092	0.2893

A (impact depth, mm), B (impact velocity, m/s), C (impact position), D (impactor diameter, mm), E (impactor material), and F (impactor shape).

**Table 3:** Range analysis for the peak maximum principal strain (MPS) in the cerebrum, hippocampus, and cerebellum.

Factors	A	B	C	D	E	F
Cerebrum						
*K*_1_	0.2550	0.3499	0.3269	0.3022	0.3141	0.4257
*K*_2_	0.4491	0.3543	0.3772	0.4019	0.3901	0.2784
*R*	0.1941	0.0044	0.0503	0.0997	0.0760	0.1473

Hippocampus						
*K*_1_	0.1741	0.2446	0.2057	0.2339	0.2388	0.3049
*K*_2_	0.3255	0.2550	0.2940	0.2657	0.2609	0.1947
*R*	0.1514	0.0104	0.0883	0.0318	0.0221	0.1102

Cerebellum						
*K*_1_	0.0904	0.1747	0.1974	0.1683	0.1540	0.2395
*K*_2_	0.2499	0.1656	0.1430	0.1721	0.1863	0.1009
*R*	0.1595	0.0091	0.0544	0.0038	0.0323	0.1386

A (impact depth), B (impact velocity), C (impact position), D (impactor diameter), E (impactor material), and F (impactor shape).
